# Influence of ultrasound on juvenile hormone titers in *Monochamus alternatus* Hope (Coleoptera: Cerambycidae)

**DOI:** 10.1038/s41598-021-81227-2

**Published:** 2021-01-14

**Authors:** Yu-Ping Zha, Xiao-Ling Wu, Zi-Yi Zhang, Jing-Yuan Chen, Qi-Cai Chen

**Affiliations:** 1 Hubei Academy of Forestry, Wuhan, 430075 People’s Republic of China; 2 College of Life Sciences and Hubei Key Lab of Genetic Regulation and Integrative Biology, Central China Normal University, Wuhan, 430079 People’s Republic of China

**Keywords:** Biochemistry, Biophysics, Physiology, Environmental sciences

## Abstract

Abiotic stress factors can significantly affect insects. In particular, the stressful effects of exposure to ultrasound on insects are considered important. In the present study, we investigated the effects of ultrasound on the important global pest *Monochamus alternatus* (Coleoptera: Cerambycidae), which is the main vector of the pinewood nematode. We exposed *M. alternatu*s adults (aged 1 day, 3 days, and 5 days) to ultrasound at different frequencies (using two ultrasonic devices, i.e., LHC20 with a mixture of frequencies at 35 kHz, 70 kHz, and 105 kHz; and GFG-8016G at two separate frequencies of 30 kHz and 60 kHz) for different periods of time (1 h, 12 h, and 24 h), before evaluating the juvenile hormone III (JHIII) titers. All of the ultrasound treatments significantly decreased the JHIII titers in *M. alternatus* adults. The decreases in the JHIII titers due to ultrasound exposure did not differ according to sex, but the effects on beetles of different ages differed significantly depending on the duration of exposure. The decreases in the JHIII titers were highest in male and female beetles after exposure to ultrasound for 12 h. Following exposure to ultrasound for any time period, the decreases in the JHIII titers were lower in adults aged 3 days than those aged 1 day and 5 days. The different ultrasonic frequencies led to variable decreases in the JHIII titers in *M. alternatus* adults, where the greatest decreases occurred in beetles exposed to ultrasound at 60 kHz. Our results indicate that ultrasound can negatively affect the normal JHIII levels and it may further disrupt sexual maturation by *M. alternatus* adults.

## Introduction

Juvenile hormone (JH) is a vital hormone that regulates reproduction, development, and behavior in insects^1–3^. JH plays primary roles in reproductive processes by stimulating or inhibiting reproductive development, although the extent and timing of the actions of JH may vary among different insects^4^. There are over four types of JH, where JHIII is the most common in Coleoptera^5^. In general, changes in the JH levels can regulate the development of insects, e.g., the JH levels are low at eclosion and higher in the adult of the mosquito *Aedes aegypti* (L.) (Diptera: Culicidae)^2^. In the locust *Locusta migratoria migratorioides* R. and F. (Orthoptera: Acrididae), JH has an important function in the longitudinal muscle developmental process at the time of oviposition in females^6^. JH is required for vitellogenin synthesis in the beetle *Tribolium castaneum* Herbst (Coleoptera: Tenebrionidae) and it remains at a high level 1–5 days after adult emergence^7^. JH is involved in mating behavior, aggregation behavior, and other behaviors in insects^1,8–10^. In particular, JH can affect the sensitivity of the olfactory antennal lobe neurons in males of the moth *Agrotis ipsilon* (Hufnagel) (Lepidoptera: Noctuidae) to modulate the timing of mate recognition^11^. Physiological evidence indicates that the JH may inhibit the activity of acetylcholinesterase in insect cells^12^.

*Monochamus alternatus* is the main vector of the pinewood nematode, *Burasphelenchus xylophilus* (Steiner and Buhrer) (Nematode: Aphelenchoididae), which causes serious damage to pines^13^. At present, there are no effective strategies for controlling *M. alternatus*, such as physical, chemical, or biological methods^14^. The application of acoustic technology in pest management is increasingly very rapidly based on techniques for producing signals that disrupt vibrational communication, as well as the development of control treatments by combining pheromones with precisely patterned sonic or vibrational signals^15^. Ultrasound (35–105 kHz) can significantly affect insects, where it can reduce the amount of eggs laid and the mating time for adult *Plodia interpunctella* (Hübner) (Lepidoptera: Pyralidae), as well as modulating the acetylcholinesterase and antioxidant enzyme systems in *Helicoverpa armigera* (Hübner) (Lepidoptera: Noctuidae)^16–18^. The ultrasound (60–80 kHz) also had a significant influence on the flight behavior of *Myrmeleon hyalinus* (Neuroptera Myrmeleontidae)^19^. *M. alternatus* is a typical nocturnal animal^20^ and it may be affected by the ultrasound produced by *Eptesicus serotinus* (Chiroptera: Vespertilionidae), which is a widespread insectivorous bat in China that mainly feeds on beetles^21^.

In our previous study, we found that ultrasound affected the acetylcholinesterase activity in *M. alternatus*^22^ and JH may play an important role in this process. To test this hypothesis, in the present study, we investigated whether exposure to ultrasound at different frequencies and times might affect the JHIII titers in *M. alternatus* adults.

## Materials and methods

### Insects

Newly infested *Pinus massoniana* bolts were collected from a forest in Songzi, Hubei, China (30° 02′ 43″ N, 111° 45′ 29″ E; 64 m above sea level). The bolts were transported to the forest entomology laboratory at Hubei Forestry Academy, Wuhan, China. The bolts were kept in a climate chamber at a temperature of 25–28 °C, relative humidity (RH) of 45–55%, and with a light:dark photoperiod of 16:8 h. Between early May and the end of June, the adult beetles that emerged from the bolts were collected each day and maintained separately in a cylindrical insect jar (diameter = 5.8 cm, height = 8.7 cm; Guangying Technology Co. Ltd, China). Fresh *P. massoniana* twigs aged one year were provided to feed the beetles every day.

### Ultrasonic devices

Two ultrasonic devices were evaluated comprising LHC20, a commercial device manufactured by Lihui, Inc., Wuhan, China^17^, and a function generator (GFG-8016G; Good Will Inst. Co. Ltd, Bayan Lepas, Penang, Malaysia). The LHC20 system could only generate one type of ultrasound with a mixture of peak frequencies at 35 kHz, 70 kHz, and 105 kHz. The GFG-8016G instrument could generate ultrasound at two frequencies of 30 kHz and 60 kHz. All of these frequencies are in the range for *E. serotinus*^23^. Ultrasound properties were measured using an ultrasound detector (D.1000X; Pettersson Elektronik AR, Sweden). Data were captured using a laptop computer equipped with a DAQCARD-AI-16E-4 acquisition card (National Instruments, Austin, Texas, USA). Data were collected at a rate of 200,000 samples/s and with 12-bit resolution. Sound pressure level (SPL) measurements were acquired based on 10 readings at any given position. SPL measurements were expressed in decibels with respect to 20 micropascals (μP; 0 dB = 20 μP).

All of the sound parameters generated by the function generator were fully programmable via a computer. The mean SPL ± standard error (SE) produced was 94.6 ± 0.3 dB at a distance of 50 cm, and the ultrasound pulse width was 500 s.

### Test procedures

*Monochamus alternatus* adults were placed individually in 100 mL plastic containers (height = 12 cm, diameter = 8 cm). The open ends of the containers were tightly covered with wire mesh screens (opening area = 30 cm^2^). The LHC20 system or the function generator were placed 50 cm above the wire mesh covering the containers. The SPLs immediately above the wire mesh were 92.1 ± 0.4 dB for the LHC20 device and 94.6 ± 0.3 dB for the function generator. The control treatments comprised separate containers with *M. alternatus* adults, which were not exposed to ultrasound.

1. Healthy beetles (aged 1–13 days) were divided into 26 groups with three adults in each group according to their sex and age. All beetles were used for extracting JHIII.
2. To test the chronic effects of ultrasound, healthy beetles were divided into 18 groups with three adults in each group according to their sex and ages (1 day, 3 days, and 5 days), and the groups were then exposed individually to ultrasound produced from the LHC20 device for periods of 1 h, 12 h, or 24 h. Each treatment was replicated three times. Each test group had a corresponding individual control group. All tests were conducted at 25 ± 1 °C, RH 65% ± 5%, and under a light:dark photoperiod of 12:12 h.
3. To test the effects of different ultrasound frequencies, healthy beetles (aged 1 day) were divided into six groups with three adults in each group according to their sex. Individuals in these groups were then exposed to ultrasound produced by the LHC20 device or function generator for 12 h. All tests were conducted at 25 ± 1 °C, RH 65% ± 5% and with a photoperiod of 12:12 h.

### Extraction and quantification of JHIII

After each treatment, individuals were cleaned with distilled water and dried using pieces of filter paper. Each sample insect was then weighed. JHIII was extracted from each sample according to the method described by Jiang^24^ with a few modifications. Each whole adult was mixed with 4 mL of methanol:ethyl ether (1:1, V/V) and homogenized in a micro-mortar using a micro-glass tube for 5 min. The mixture was transferred to 10 mL centrifugal tubes and sonicated for 5 min with a UP200S ultrasonic processor (Hielscher, Germany), before adding 2 mL of n-hexane, sonicating again for 5 min, and centrifuging at 4000 r/min and 4 °C for 10 min. The upper n-hexane phase was collected and the lower layer was subjected to ultrasound-assisted re-extraction five times where 2 mL of n-hexane was added after each sonication treatment. At the end of each centrifugation step, the n-hexane phases were mixed together. The combined n-hexane extracts were dried under nitrogen and dissolved in 500 μL methanol, before filtering through a 0.22 μm Millipore Express PES Membrane, and storing at − 20 °C until their analysis.

The JHIII titers were determined by high-performance liquid chromatography (HPLC) using a DGU-20A5 (Japan) system equipped with a Luna 5 μm C18 column (250 mm × 4.6 mm) and a flow-through ultraviolet (UV) detector. JHIII was eluted with 80% methanol in water at a flow rate of 1 mL/min with UV detector monitoring at 220 nm. The injection volume was 30 μL and the column temperature was set at 30 °C. JHIII titers were quantified by comparing the peak areas with those obtained for external quantitative standards^24,25^. Each sample was analyzed three times by HPLC to ensure precision, and the average was used as the JHIII titer for a single sample at a given time point.

We identified and quantified JHIII by reverse phase HPLC. JHIII was identified based on its retention time relative to the JHIII standard but we could not exclude the possibility that other organic compounds might co-elute and absorb at the same wavelength as JHIII. Therefore, the JHIII titers might have been lower than those determined in this study^25^.

### Data analysis

The decrease in the JHIII titer was calculated as the JHIII titer in the control group minus the JHIII titer in the test group divided by the JHIII titer in the control group and multiplied by 100%. All data were analyzed by one-way analysis of variance using GraphPad Prism (version 4.0 for Mac, GraphPad Software, San Diego, California, USA). The results were expressed as the mean ± SE. Tukey’s multiple comparison test was performed when significance differences were detected at *P* < 0.05.

## Results

**Figure 1:**
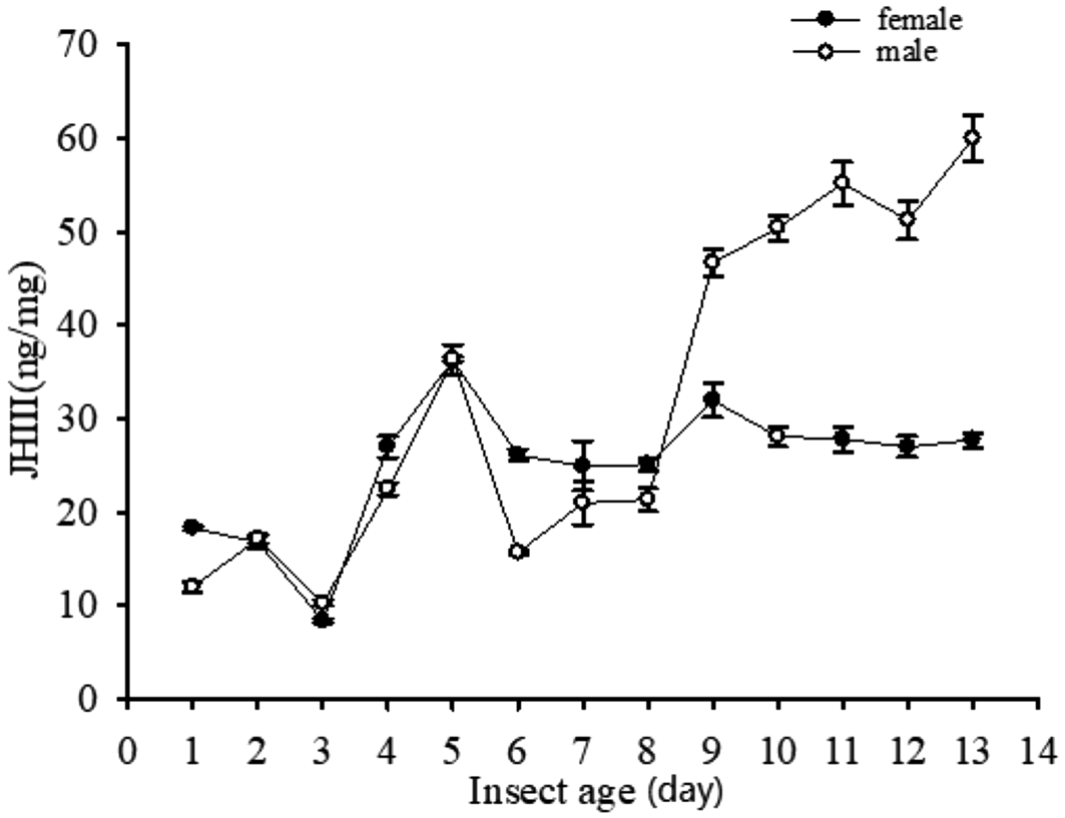
Changes in the juvenile hormone III (JHIII) titer in the absence of ultrasound in *Monochamus alternatus* adults. Each point represents the mean ± SE analyzed by HPLC and each HPLC measurement was repeated three times for a single sample to validate the analytical precision. Total number of *M. alternatus* adults across all three replicates: n = 234.

In this study, we measured the JHIII titers in *M. alternatus* adults (Fig. 1). In the absence of ultrasound, we found that the JHIII titers in *M. alternatus* adults differed according to sex. On the day of emergence, the JHIII titer in female adults was only slightly higher than that in males. On days 1–2, the JHIII titers were similar in both sexes. From day 3 to day 5, the JHIII titers increased sharply in females and males. Female adults had the highest titer after 5 days but the titer then decreased gradually over the next 8 days. The JHIII titer in males decreased on day 6, but then increased slightly until 13 days.

**Figure 2:**
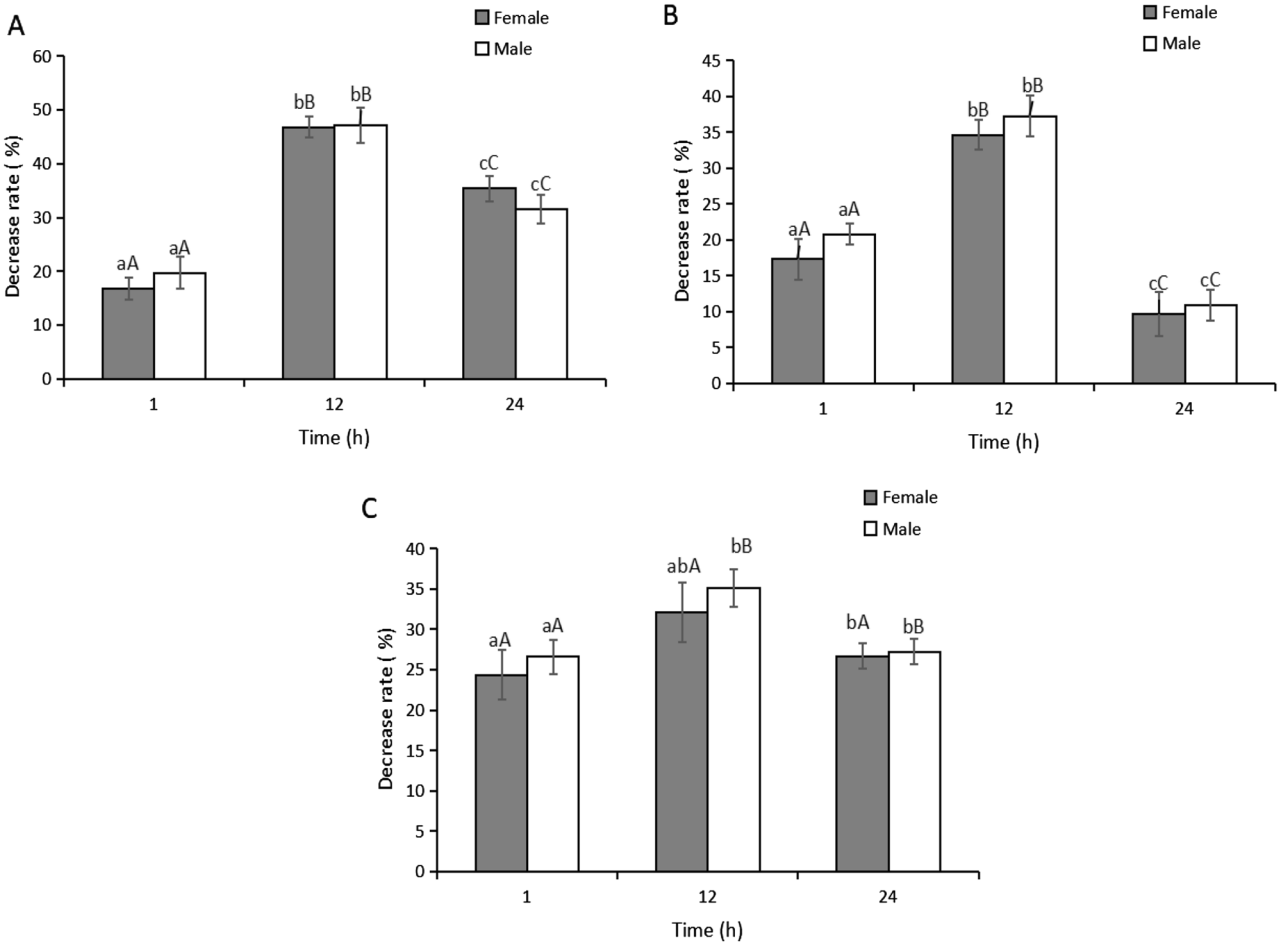
Decrease rate of juvenile hormone III titers of *Monochamus alternatus* adults during different period of time in the presence of ultrasound of LHC20. Bars are the mean ± SE for n = 324 of total numbers of *M. alternatus* adults across all three replicates. Different letters with the different bars indicate significant differences at the 5% and 1% level. (**A**) 1-day-old *M. alternatus* adults; (**B**) 3-day-old *M. alternatus* adults; (**C**) 5-day-old *M. alternatus* adults.

When the beetles were exposed to ultrasound produced from the LHC20 system for different periods, we found that the decreases in the JHIII titers varied significantly (Fig. 2), but not significantly between males and females. For the 1-day-old female and male adults, highly significant differences (*P* < 0.01) were found in the mean decreases in the JHIII titers after ultrasound treatment for 1 h, 12 h, and 24 h. For the 3-day-old female and male adults, the mean (± SE) decreases in the JHIII titers after ultrasonic treatment for 12 h were 34.61% ± 2.12% and 37.26% ± 2.78%, respectively, which were significantly higher (*P* < 0.01) than those after exposure times of 1 h and 24 h. In 5-day-old female and male adults, the mean (± SE) decreases in the JHIII titers after ultrasonic treatment for 12 h were 32.14% ± 3.71% and 35.08% ± 2.33%, respectively, which were also significantly higher (*P* < 0.05) than those after exposure for 1 h and 24 h. Thus, the greatest decreases in the JHIII titers in *M. alternatus* adults occurred after exposure to ultrasound for 12 h.

**Figure 3:**
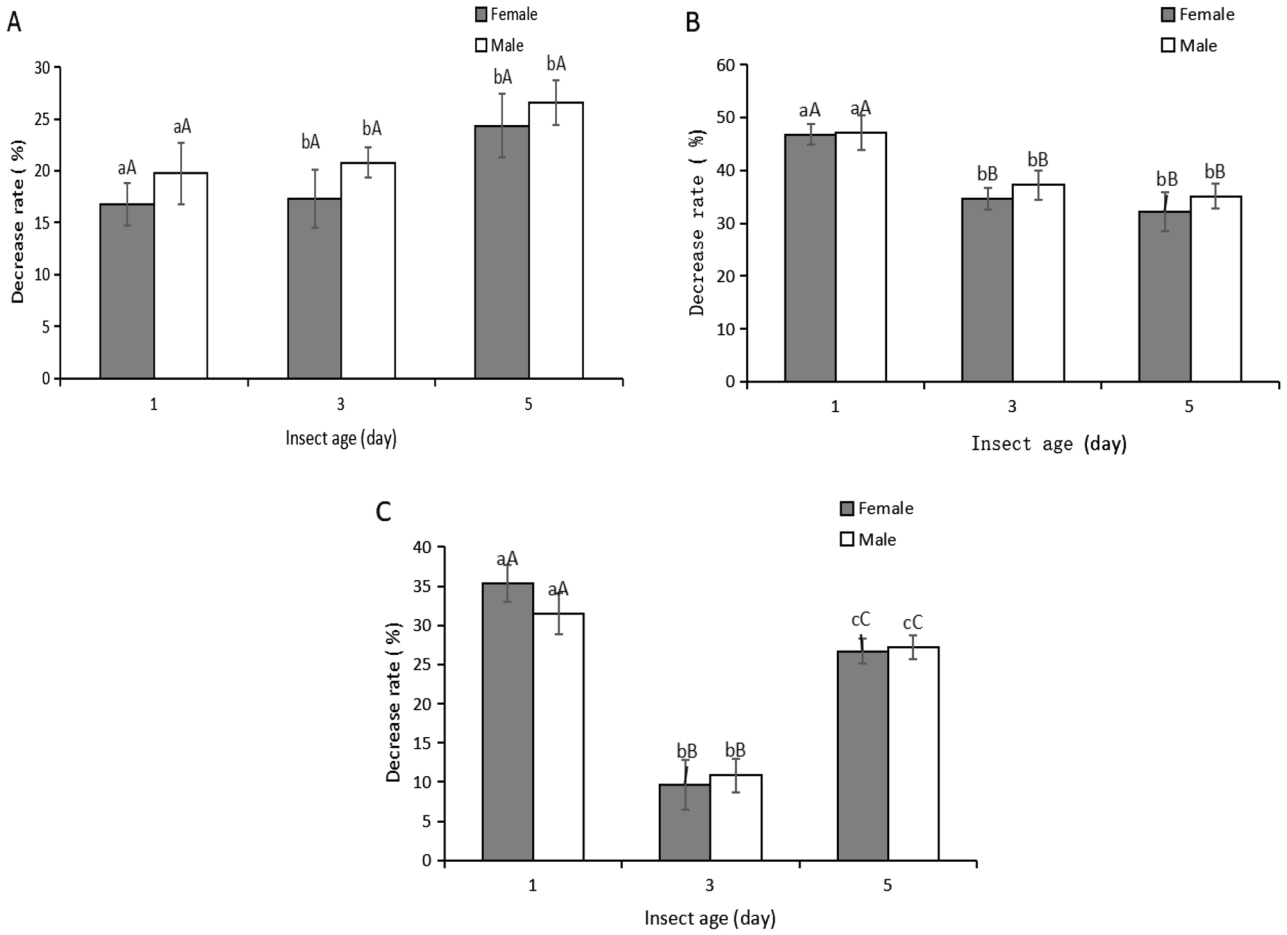
Decrease rate of juvenile hormone III titers of *Monochamus alternatus* adults with difference ages in the presence of ultrasound of LHC20. Bars are the mean ± SE for n = 324 of total numbers of M. alternatus adults across all three replicates. Different letters with the different bars indicate significant differences at the 5% and 1% level. (**A**) 1 h ultrasound exposed; (**B**) 12 h ultrasound exposed; (**C**) 24 h ultrasound exposed.

We also found that the decreases in the JHIII titers varied significantly (Fig. 3) when beetles with different ages were exposed to ultrasound produced using the LHC20 system for the same amount of time. After ultrasonic treatment for 1 h and 12 h, the mean (± SE) decreases in the JHIII titers were significantly higher in the 5-day-old adults (*P* < 0.05) than the 1-day-old or 3-day-old adults. However, after ultrasonic treatment for 24 h, the mean (± SE) decreases in the JHIII titers were significantly lower in 3-day-old adults (*P* < 0.01) than 1-day-old or 5-day-old adults. These results indicated that ultrasonic treatment had different stressful effects on the endocrine regulation system in *M. alternatus* adults depending on their age.

**Figure 4:**
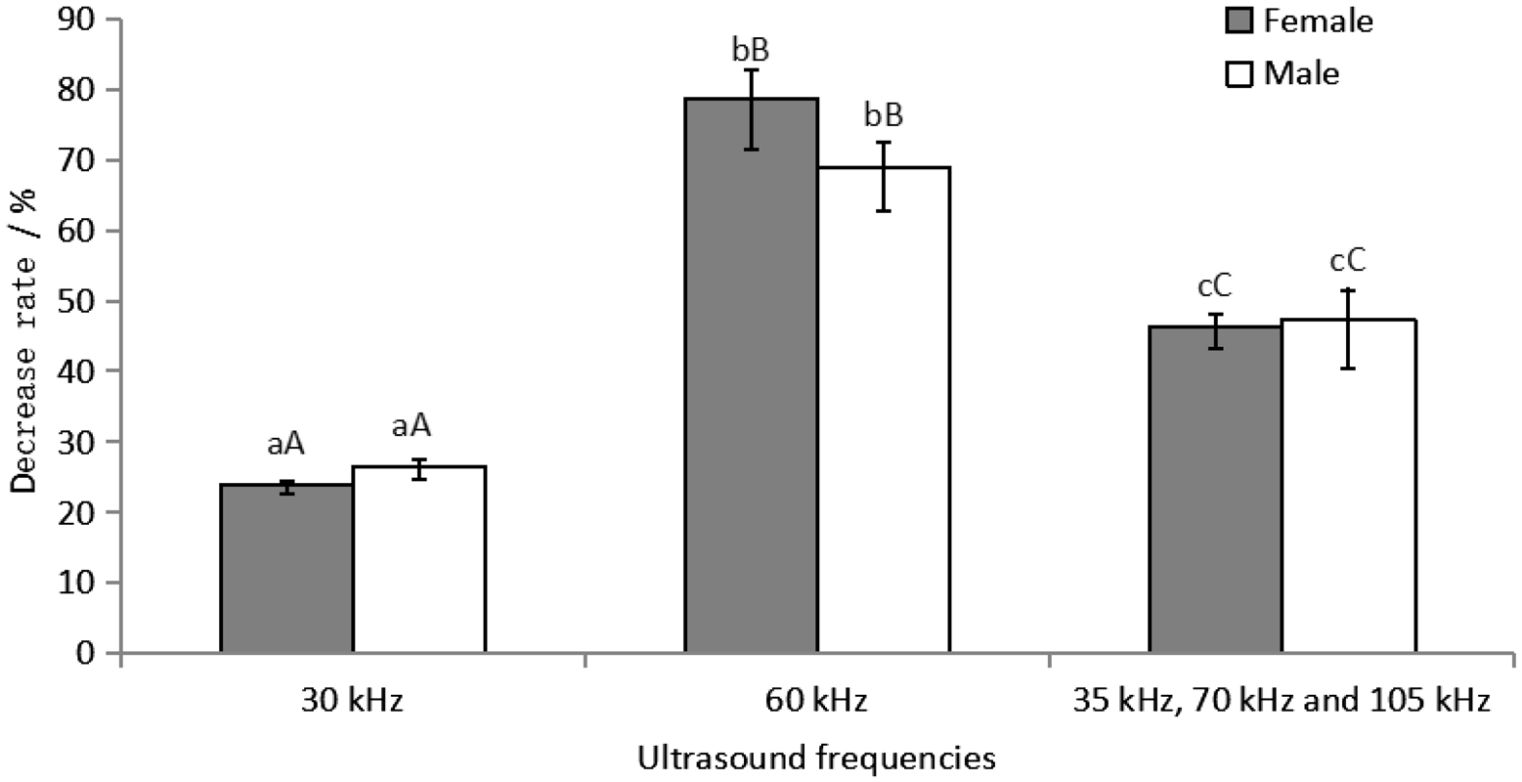
Decreases in juvenile hormone III titers in *Monochamus alternatus* adults after exposure to ultrasound at different frequencies. Different letters on bars indicate significant differences at *P* < 0.05. Data obtained for the same exposure time were analyzed by one-way analysis of variance, and the least significant difference test was used to compare means. Bars represent the mean ± SE for n = 108 *M. alternatus* adults across all three replicates.

Ultrasound production by both devices significantly reduced the JHIII titers in *M. alternatus* adults, but there were no significant differences between males and females (Fig. 4). The decreases in the JHIII titers in male beetles were 23.86% ± 1.20%, 78.64% ± 7.13%, and 46.25% ± 3.04%, whereas those in female beetles were 26.46% ± 1.84%, 68.87% ± 6.16%, and 47.35% ± 7.05% after exposure to ultrasound generated by the function generator or LCH20 for 12 h, respectively. Ultrasound at a frequency of 60 kHz resulted in the greatest decrease in the JHIII titers (*P* < 0.01) compared with the other ultrasound frequencies.

## Discussion

JH plays various roles in developmental and physiological processes in adult insects^26–28^. In particular, changes in the JH titer are essential for reproductive maturation in insects. After emergence, the JH titer increases significantly from the 7th day in *L. migratoria* females^6^, whereas it increases gradually from the 11th day in *Schistocerca gregaria* (Forskai) (Orthoptera: Acrididae) males^29^, the similar trend was found in *Onthophagus taurus* (Coleoptera: Scarabaeidae)^30^. In our study, the JH titers in *M. alternatus* were low at eclosion and they increased from the third day after adult emergence. Female adults had the highest titers when they were 5 days old, but they then decreased gradually over the next 8 days. The JHIII titers in males decreased on day 6, but then increased slightly until day 13 (Fig. 1). These results may help to explain why 5-day-old males can copulate successfully and 6-day-old females can oviposit eggs^31^.

Various changes in the JH titers occur in both sexes in *M. alternatus* adults, and we consider that these changes are necessary for oviposition based previous studies^6,7,31^. The differences in the JH titers of *M. alternatus* males and females are probably important for the development of the sexes. The rates of maturation and reproductive behavior also differ in *M. alternatus* males and females. The JH levels increase after emergence and initiate vitellogenesis, mating, and other processes^32^.

The JH titers can vary according to various internal (e.g., hormonal and genetic) and external (e.g., temperature and nutritional) factors^33,34^. The photoperiod and temperature may greatly influence the biosynthesis of JH and sexual maturation in the cotton bollworm^35^.

Sound stress is generally considered to be an environmental stress with severe impacts on animals and humans. Ultrasound produced from the LHC20 device was perceived as a sound stress by *M. alternatus*. Compared with the control group, we found that the JH titers decreased significantly under ultrasound treatment (Figs. 2 and 3), and these results are consistent with our previous studies^17,22^. Ultrasound stress can significantly influence the activity of acetylcholinesterase in *H. armigera*^17^. The cholinergic system in *M. alternatus* adults is also modulated by ultrasound stress^22^. Similar effects of ultrasound stress on insects were observed in other studies^16,36–38^. These results suggest that ultrasound can produce an environmental stress effect on *M. alternatus* and act as an external stimulatory factor that might affect the hormone metabolism system in adults.

In the present study, we compared the JHIII titers in *M. alternatus* adults exposed to ultrasound at different frequencies (Fig. 4). We found that exposure to ultrasound at a frequency of 60 kHz reduced the JHIII titers to the lowest level and this frequency is in the range of predatory bats. Thus, the ultrasound frequencies produced by bats might influence the physiological activities of insects^39^. Our results showed that different ultrasound frequencies, especially bat-like frequencies, could significantly decrease the JHIII titers in *M. alternatus* adults, thereby disrupting the normal JHIII titers.

Many previous studies of JH metabolism have focused on JH esterases^40^. The regulation of JH production in adult insects is a complex process that involves endogenous neuroendocrine signals^41^. Our results suggest that ultrasound might disrupt the balance of JH synthesis and degradation, but identifying the specific molecular mechanisms involved requires further study (“Supplementary Information”).

## Conclusion

In the present study, we demonstrated that exposure to ultrasound significantly decreased the JHIII titers in *M. alternatus* adults. The decreases in the JHIII titers due to exposure to ultrasound did not differ between the sexes, but the effects on beetles with different ages varied significantly depending on the duration of exposure. The decreases in the JHIII titers were highest in male and female beetles after exposure to ultrasound for 12 h. Different ultrasound frequencies had variable effects on the decreases in the JHIII titers in *M. alternatus* adults. Exposing beetles to ultrasound at a frequency of 60 kHz resulted in the greatest decrease in the JHIII titers.

## Supplementary Information

Supplementary Information.
