# Supplementary material for: Influence of ultrasound on juvenile hormone titers in Monochamus alternatus Hope (Coleoptera: Cerambycidae)
Source: Sci Rep. 2021 Jan 14;11:1450. doi: 10.1038/s41598-021-81227-2 (PMC7809024; doi:10.1038/s41598-021-81227-2)

**Influence of ultrasound on juvenile hormone titers in *Monochamus alternatus* Hope (Coleoptera: Cerambycidae)**

**Yu-Ping Zha·Xiao-Ling Wu·Zi-Yi Zhang·Jing-Yuan Chen·Qi-Cai Chen**

**Support matieral**

1. The commercial device, LHC20, is used for arthropod pest managements. LHC20: Input voltage: 220 V ± 22 V; Frequency: 50 Hz ± 1 Hz; Power: 7 W; Volume: 125 mm ×78 mm ×45 mm; Weight: 220 g.


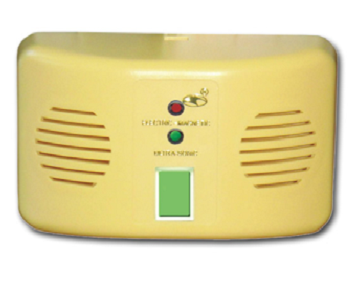


1. Sound measurements were made at a distance of 50 cm from the ultrasonic device’s transducer, with the device running in “on” setting. Measurements were made using D1000X Bat detector (Pettersson Elektronik, Sweden). Sound measurements included peak frequencies and sound pressure levels. Data were analyzed using Raven Pro 1.3 (Cornell Lab of Ornithology, USA). The device used in the tests generated peak frequencies at 33 and 69 kHz (Fig. 1). And the device produced a 97 dB SPL at a distance of 50 cm from the source. The waveform plot (Fig. 2) showed the ultrasound pulse width was 0.02 s.


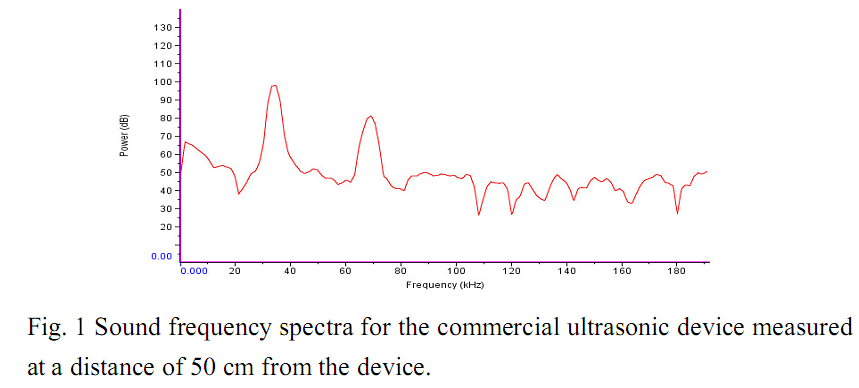


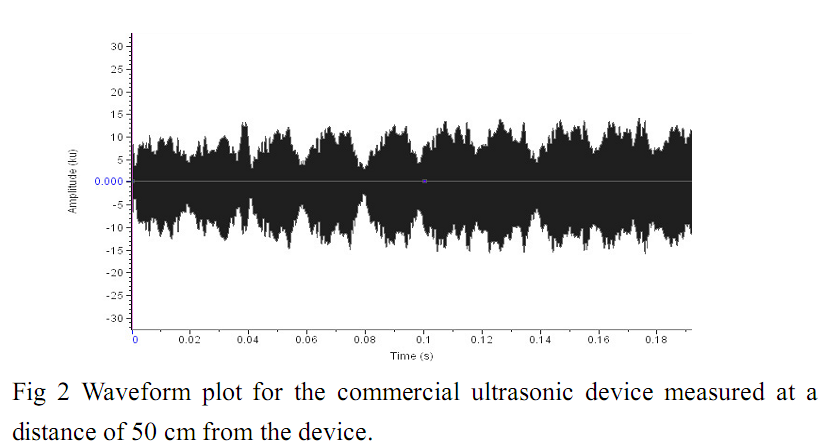

Supplement: Supplementary file 1 — Supplementary Information. [file 41598_2021_81227_MOESM1_ESM.doc]
